# Construction of a Suite of Computable Biological Network Models Focused on Mucociliary Clearance in the Respiratory Tract

**DOI:** 10.3389/fgene.2019.00087

**Published:** 2019-02-15

**Authors:** Hasmik Yepiskoposyan, Marja Talikka, Stefano Vavassori, Florian Martin, Alain Sewer, Sylvain Gubian, Karsta Luettich, Manuel Claude Peitsch, Julia Hoeng

**Affiliations:** PMI R&D, Philip Morris Products S.A., Neuchâtel, Switzerland

**Keywords:** mucociliary clearance, network models, biological expression language, respiratory tract, network perturbation amplitude

## Abstract

Mucociliary clearance (MCC), considered as a collaboration of mucus secreted from goblet cells, the airway surface liquid layer, and the beating of cilia of ciliated cells, is the airways’ defense system against airborne contaminants. Because the process is well described at the molecular level, we gathered the available information into a suite of comprehensive causal biological network (CBN) models. The suite consists of three independent models that represent (1) cilium assembly, (2) ciliary beating, and (3) goblet cell hyperplasia/metaplasia and that were built in the Biological Expression Language, which is both human-readable and computable. The network analysis of highly connected nodes and pathways demonstrated that the relevant biology was captured in the MCC models. We also show the scoring of transcriptomic data onto these network models and demonstrate that the models capture the perturbation in each dataset accurately. This work is a continuation of our approach to use computational biological network models and mathematical algorithms that allow for the interpretation of high-throughput molecular datasets in the context of known biology. The MCC network model suite can be a valuable tool in personalized medicine to further understand heterogeneity and individual drug responses in complex respiratory diseases.

## Introduction

The respiratory tract is under constant challenge to provide the body with oxygen while monitoring air quality for pollutants and microorganisms. The mucous membranes in the airways, which are lined with microtubule-based projections, the cilia, represent a powerful first-line defense. In response to irritants and infection, mucus is secreted by goblet cells, and cilia on the surface of ciliated cells move mucus upward in coordinated waving and beating motions. Eventually, particles are expelled through sneeze and cough (Wanner et al., 1996). This self-clearing mechanism, mucociliary clearance (MCC), ensures proper functioning of the respiratory tract.

Cilia have attracted increasing attention because of the growing number of diseases caused by mutations in genes that impact cilium assembly, function, and turnover (Fliegauf et al., 2007; Kempeneers and Chilvers, 2018). Traditionally, cilia are classified as primary or motile (Wheatley, 1995; Satir and Christensen, 2007). Primary cilia are present on almost all cell types and are involved in tissue homeostasis (Gerdes et al., 2009; Nozawa et al., 2013). Motile cilia often occur as clusters of several hundred protrusions covering cells and direct fluid flow (Choksi et al., 2014).

Cilia assembly and resorption often depend on the cell cycle (Kim and Tsiokas, 2011), with a neatly interwoven mode of regulation assuring timely and developmentally precise control of cilium biogenesis. The regulatory factor X (RFX) family of transcription factors is a key regulator of both primary and motile cilia assembly programs (reviewed in Thomas et al., 2010; Choksi et al., 2014). A master regulator of motile cilia assembly across the vertebrates is forkhead box J (FOXJ1), a member of the forkhead/winged-helix family of transcription factors (Murphy et al., 1997; Chen et al., 1998; Brody et al., 2000), which is under control of multiciliate differentiation and DNA synthesis-associated cell cycle protein (MCIDAS) and geminin coiled-coil domain-containing (GMNC) protein in the respiratory epithelium (Stubbs et al., 2012; Arbi et al., 2016). Mutations in MCIDAS and its downstream effector cyclin O are implicated in an MCC disorder known as reduced generation of multiple motile cilia (RGMC) (Boon et al., 2014). In RGMC patients, cilia numbers are reduced, resulting in impaired MCC, airway obstruction, and recurring respiratory infections.

An alternative mechanism of ciliary regulation is the disassembly of the organelle by aurora A kinase (AURKA), which also regulates the entry into mitosis (Pan et al., 2004; Pugacheva et al., 2007). AURKA phosphorylates histone deacetylase 6 (HDAC6), stimulating HDAC6-dependent deacetylation of axonemal microtubules (Hubbert et al., 2002), destabilization of the ciliary shaft, and subsequent collapse of the cilium.

Exposure to air pollutants, cigarette smoke, drugs, or infectious agents can affect ciliary beating frequency (CBF) (Workman and Cohen, 2014; Yaghi and Dolovich, 2016). On the molecular level, CBF increases in response to high mucus viscosity (Fernandes et al., 2008) and fluctuations in the levels of second messengers, such as cyclic adenosine 3′,5′-mono- phosphate (cAMP), cyclic guanidine 3′,5′-mono- phosphate (cGMP), intracellular Ca^2+^, calmodulin, nitric oxide (Jain et al., 1993; Korngreen and Priel, 1996; Yang et al., 1996; Wyatt et al., 1998; Zagoory et al., 2001, 2002), and intracellular pH (Sutto et al., 2004). Mechanistically, CBF increases as a result of cAMP- and cGMP-mediated activation of respective protein kinases via Ca^2+^ release or by a calcium-independent mechanism.

While mucus secretion is a normal defense response, mucin synthesis in goblet cells and mucus secretion are amplified in respiratory diseases such as asthma or chronic obstructive pulmonary disease (COPD). In addition, the number of goblet cells can increase by proliferation (hyperplasia) and by airway epithelial cell transdifferentiation (metaplasia), further contributing to increased mucus production (Blyth et al., 1998; Rogers, 2007; Turner and Jones, 2009; Boucherat et al., 2013; Ramos et al., 2014). This airway epithelial remodeling decreases ciliated cell numbers and ciliary beating efficiency, reducing MCC and aggravating airway plugging (Nini et al., 2012; Yaghi et al., 2012; Yaghi and Dolovich, 2016).

There is overwhelming evidence that oxidative stress and oxidative damage play a pivotal role in the pathogenesis of COPD (Rahman and MacNee, 1999; Rahman and Adcock, 2006; Anderson and Macnee, 2009; Kim and Criner, 2015; Matera et al., 2016). Oxidative stress is a well-described trigger of the epidermal growth factor receptor (EGFR) signaling pathway that leads to mucus hypersecretion (Takeyama et al., 1999, 2001; Perrais et al., 2002; Hewson et al., 2004; Casalino-Matsuda et al., 2006; Hao et al., 2014). We recently published an adverse outcome pathway that describes the events that follow oxidative stress-mediated EGFR activation to goblet cell hyperplasia/metaplasia and decreased lung function following mucus overproduction (Luettich et al., 2017).

Signaling downstream of interleukin (IL) 13 is involved in the pathogenesis of asthma (Wills-Karp, 2004). The IL13 receptor complex initiates several cascades of molecular events that result in goblet cell metaplasia/hyperplasia. One important downstream effector of IL13 is the sterile alpha motif pointed domain-containing ETS transcription factor (SPDEF), which is directly involved in mucin gene expression (Park et al., 2007; Chen et al., 2009).

The vast volume and diversity of biological data available on cilium assembly, CBF, and goblet cell hyperplasia/metaplasia require that the information be integrated for better visualization and understanding of the processes that underlie respiratory diseases. Biological network models offer a framework for understanding biological processes and diseases and aid in drawing new, often unpredicted conclusions. Over the years, we have built several causal biological network (CBN) models that capture biological processes that are impacted in COPD. These models, stored in the CBN database, are emerging as an innovative and powerful tool to quantify the impact of exposure or potentially affected biological processes in disease (Cho et al., 2012; Martin et al., 2014; Boue et al., 2015; Talikka et al., 2017). The major advantage of the CBN approach is that it transforms unstructured data into interconnected and organized knowledge that describes biological processes precisely and accurately (Schlage et al., 2011; Westra et al., 2011, 2013; Cho et al., 2012; Gebel et al., 2013; De Leon et al., 2014; Martin et al., 2014; Szostak et al., 2016).

In this study, we present a suite of causal biological models that describe important molecular events involved in MCC, from cilium assembly to ciliary beating, goblet cell hyperplasia/metaplasia, and mucus hypersecretion. We also show how transcriptomic data are scored onto these network models and how the models can provide mechanistic understanding of gene expression changes.

## Materials and Methods

### Literature Curation

Biological Expression Language (BEL)^1^ version 1.0 is used for scientific text curation. BEL is a computable language that converts causal and correlative biological observations to statements consisting of two biological entities connected by a relationship predicate^1^. Relevant original research articles for curation were identified from pertinent review articles in the field. The journal impact factor or any other means to rank the publications was not considered. If the statements in the original research articles were sufficiently supported by the results presented in figures, the information was considered reliable and captured. To retrieve causal relationships the result sections were extracted from these articles for curation. The introduction, discussion and conclusion sections were avoided because the evidences therein largely contain data from earlier studies, repetition of the results, hypotheses and assumptions. Although several evidences supporting an interaction would provide more confidence on the edge, we capture the interactions even when a single experiment is provided in the literature, in order to not omit the relevant information. Contradicting statements were captured without preferential treatment and with proper annotations (model organism, tissue, cell line, treatment/disease, experimental setup). The experimental information from the relevant peer-reviewed scientific articles is semi-automatically processed through the BEL Information Extraction workFlow (BELIEF) platform (Szostak et al., 2015, 2016). BELIEF contains a text-mining software that recognizes biological terms in the text and assembles them into BEL statements. The curation interface allows review, correction, and annotations (cell/tissue type, disease if applicable, species, and experimental design) of the statements that BELIEF proposes. The literature curation is an iterative process. After the curation of initial articles, a gap analysis is performed, and more literature is identified based on gaps in the network models.

### Network Model Assembly and Visualization

BEL statements are then compiled to generate a cohesive knowledge assembly model using the OpenBEL framework 3.0.0, an open source compilation framework. The network model consists of nodes that are the biological entities in the network models connected by edges (i.e., the relationships between the biological entities). Any RNA nodes are removed from the model backbone and used in the downstream layer for model scoring as described in Martin et al. (2014). The Cytoscape web application^2^ is used to visualize and analyze the network properties (Shannon et al., 2003). Cytoscape supports powerful visual mapping whereby biological entities are depicted as defined-shaped nodes connected by the relationship edges. The network visualization is used also during the curation process to identify the gaps and to trim the network models. The trimming here means that any nodes that are “hanging” and do not lead to a biological process described in the model are removed, or further curation is performed to add molecular relationships to connect such nodes to the biological process.

The network model suite is available in the CBN database. The NPA algorithm as well as some measurable “downstream” relationships (backbone node to mRNA) can be downloaded as R packages from the GitHub project pages https://github.com/pmpsa-hpc/NPA and https://github.com/pmpsa-hpc/NPAModels.

### Network Model Scoring

The network perturbation amplitude (NPA) methodology is used to obtain a quantitative assessment of how each of the models interprets the transcriptomic changes in the datasets we selected (GSE22430, GSE37693, and GSE5264). This methodology allows for the translation of gene expression fold-changes to differential values for each network node as well as enabling a network-level summary to provide a quantitation of the degree of network model perturbation (Hoeng et al., 2012; Martin et al., 2014; Sewer et al., 2015; Szostak et al., 2016). Raw data were obtained from Gene Expression Omnibus (GEO) repository and normalized following a standard pipeline based on robust multiarray normalization implemented in the R environment for statistical computing (Smyth, 2004). The differential expression values and statistics were calculated using the Bioconductor LIMMA package with appropriate experimental comparisons. “O” and “K” statistics was used to test the specificity of the network models (including the “downstream edges” that connect the network nodes to gene differential expression nodes according to the underlying reverse-causal concept; Catlett et al., 2013). They compare the actual NPA value to the distributions of alternative NPA values obtained by permuting the edges of the networks (the connections between nodes for “K” and the connection between nodes and gene differential expression nodes for “O”). If the actual NPA value is significantly different from these “background” non-biological values, then we consider it as significantly specific.

The leading node analysis allows to focus on a fewer number of nodes in the network by ranking the nodes based on their contribution (%). Using an empirical 80% collective contribution instead of the actual rank, does not limit the number of the nodes, when the contribution of several nodes is almost equal (Martin et al., 2014).

## Results

### Model Description

#### Cilium Assembly Model

**FIGURE 1:**
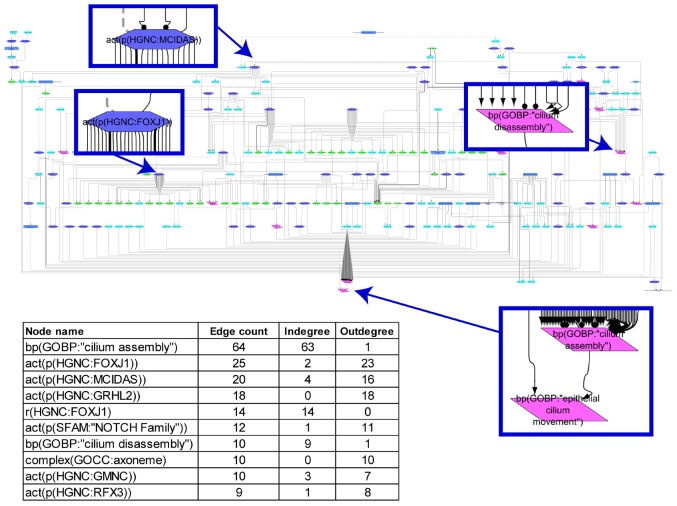
Causal biological network model for cilium assembly. The table shows the top 10 highly connected nodes and their degrees of distribution. The vocabulary for the BEL is provided in http://www.openbel.org/. The Cytoscape layout is the Yfiles hierarchical layout. The network model can be downloaded from causalbionet.com.

The cilium assembly network model is a collection of intertwined biological entities and processes that are supported by 59 relevant peer-reviewed articles. The network contains 209 nodes and 319 edges that represent relationships between nodes (Figure 1). When the connections between the nodes in the network were analyzed, many poorly connected nodes and a few highly connected ones, “hubs,” were observed. The most connected node (63 indegree edges) was the biological process “cilium assembly,” and the transcription factor FOXJ1, which is downstream of MCIDAS and GMNC, had the most outdegree edges (Figure 1).

A number of pathways, including delta-like canonical notch ligand (DLL)/NOTCH (through MCIDAS/GMNC), smoothened/hedgehog, and grainyhead-like transcription factor 2, converge into a FOXJ1/RFX module that triggers cilium assembly in the network model. This shows a high level of cooperativity between FOXJ1 and RFX factors; FOXJ1 can induce RFX2 and RFX3 expression, FOXJ1 gene expression is partially dependent on RFX3 activity, and a subset of FOXJ1 and RFX target genes overlap (Figure 1). This assures timely and developmentally precise control of cilium biogenesis. In addition, numerous molecules and complexes necessary for structural integrity of cilia, such as the axoneme constituents, BBSome complex (structural components of the basal body), and exocyst complex (membrane transport to cilium), support the “cilium assembly” hub as immediate neighbor nodes. With regard to the “cilium disassembly” hub, as expected, the AURKA-HDAC6 axis and their upstream regulators emerged as a supporting subnetwork.

#### Ciliary Beating Model

**FIGURE 2:**
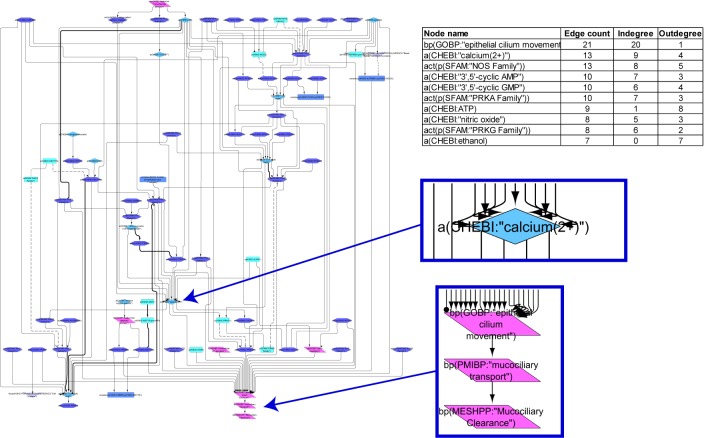
Causal biological network model for ciliary beating. The table shows the top 10 highly connected nodes and their degrees of distribution. The vocabulary for the BEL is provided in http://www.openbel.org/. The Cytoscape layout is the Yfiles hierarchical layout. The network model can be downloaded from causalbionet.com.

The ciliary beating network model was computed from 52 articles and comprises 80 nodes and 137 edges. The network illustrates the path from various stimuli through intermediate signaling molecules converging into consecutive biological processes, with “mucociliary clearance” as the final node (Figure 2). “Epithelial cilium movement” has the most inward connections in the network, and adenosine triphosphate has the most outward connections. Calcium and “nitric oxide synthase family” are central hubs in the network, with several incoming and outgoing edges. The model shows the CBF increases as a result of cAMP- and cGMP-mediated activation of the respective protein kinases through Ca^2+^ release or by a calcium-independent mechanism. The model also captures cystic fibrosis transmembrane conductance regulator, whose activation triggers the adenylate cyclase (ADCY)/cAMP pathway. Several other stimuli, such as serotonin or macrophage-stimulating protein, via corresponding receptors (HTR and MST1R, respectively), lead to increased ciliary motion in the model. Another level of regulation is added through sex hormone-dependent modulation, such as progesterone-mediated decreases or estrogen-mediated increases in CBF.

#### Goblet Cell Hyperplasia/Metaplasia Model

**FIGURE 3:**
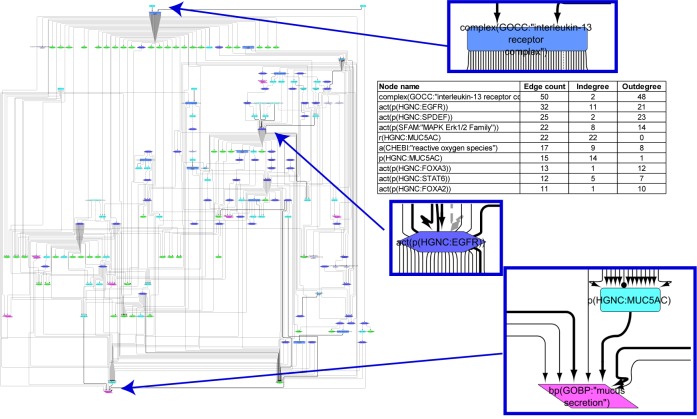
Causal biological network model for goblet cell hyperplasia/metaplasia. The table shows the top 10 highly connected nodes and their degrees of distribution. The vocabulary for the BEL is provided in http://www.openbel.org/. The Cytoscape layout is the Yfiles hierarchical layout. The network model can be downloaded from causalbionet.com.

The goblet cell hyperplasia/metaplasia model covers 172 nodes and 335 edges that were obtained from 58 articles. The hierarchical view of the network model clearly indicates that, as expected, the biological process “mucus secretion” is the endpoint of the model (Figure 3). The network model hinges on EGFR and IL signaling pathways (Figure 3). An array of growth factors such as epidermal growth factor, transforming growth factor, tumor necrosis factor, amphiregulin, IL4, IL6, IL7, IL8, and IL13 initiate goblet cell-specific mucus secretion by activating their respective receptors (EGFR and IL6R, IL13R, IL17R) and subsequent signaling events, notably through Ras/Raf/mitogen-activated protein kinase kinase/mitogen-activated protein kinase (MAPK)/extracellular signal-regulated kinase 1/2 (ERK1/2) or janus kinase/signal transducer and activator of transcription/SPDEF effectors, modulating mucin gene expression. Multiple additional factors leading to mucus hypersecretion and their interactions are also depicted in the network model. FOXA2 transcription factor, in contrast, limits goblet cell differentiation in the lung and directly represses mucin gene expression. The network displays the inhibition of FOXA2 by EGFR and IL13 pathways that results in goblet cell hyperplasia and mucus secretion.

### Model Scoring With Transcriptomic Data

#### NPA

Network scoring with transcriptomic data is based on the inference of activities of the molecular entities in the network from gene expression changes. This backward reasoning employs a downstream layer with information on gene expression changes known to be induced by the backbone entities (Martin et al., 2014). To test the ability of the MCC network models to provide a quantitative measure of MCC, we identified publicly available datasets in Gene Expression Omnibus. The first dataset selected for model scoring (GSE22430) was from lung epithelial cells treated with the redox-active toxin pyocyanin from *Pseudomonas aeruginosa* that stimulates EGFR (Rada et al., 2011). Dataset GSE5264 was derived from an *in vitro* experiment, in which airway epithelial cells were allowed to differentiate to a pseudostratified epithelium at the air-liquid interface (Ross et al., 2007). Finally, we used a transcriptomic dataset from IL13-treated human airway epithelial cells (GSE37693) (Alevy et al., 2012).

**FIGURE 4:**
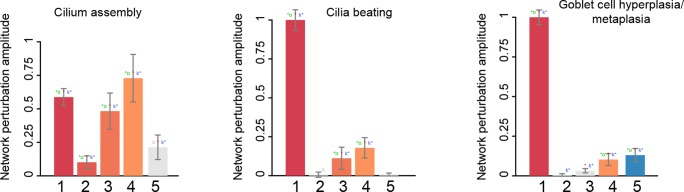
The network perturbation amplitude (NPA). The NPA scores are shown with their confidence interval, accounting for experimental variation. The red star indicates that NPA is statistically different from 0. In addition, companion statistics derived to inform on the specificity of the NPA score with respect to the network structure are shown as ^∗^O and K^∗^, respectively, if their *p*-values are below the significance level of 0.05, and by O and K when the corresponding *p*-values are between 0.05 and 0.1. ^∗^ indicates O and K statistic *p*-values below 0.05 (in color), O and K *p*-values between 0.05 and 0.1 (in gray). Lanes: 1. GSE22430, pyocyanin-treated vs. control; 2. GSE5264, early time points of mucociliary differentiation in human airway epithelial cells; 3. GSE5264, intermediate time points of mucociliary differentiation in human airway epithelial cells; 4. GSE5264, late time points of mucociliary differentiation in human airway epithelial cells; 5. GSE37693, IL13-treated vs. control.

The cilium assembly network model responded strongly to the treatment of lung cells with pyocyanin and to the time-course of bronchial epithelial cell differentiation with increasing amplitude over time. There was no impact on the models in response to the IL13 treatment (Figure 4). When the same datasets were used to score the cilia beating network models, the largest amplitude of network perturbation was observed in response to pyocyanin treatment of lung cells (Figure 4). Similar to the cilium assembly model, the amplitude of cilia beating network perturbation increased with advanced mucociliary differentiation, and the model did not respond to the IL13 treatment. The scoring of the goblet cell hyperplasia/metaplasia network model again showed a very strong response to the pyocyanin treatment and, to a lesser extent, to mucociliary differentiation of airway cells. This model responded to IL13 treatment (Figure 4).

#### Leading Node Analysis

To investigate the mechanistic foundation underlying the perturbations of the network models from transcriptomic data and to further validate the biology in the models, we used the leading node analysis (Martin et al., 2014). Leading nodes are the entities in the network models upon which the impact contributes 80% of the observed effect on the network as a whole. Leading node analysis also allows for the assessment of the directionality (activation or inhibition) of the inferred effect on each node. All leading nodes for all contrasts and models are provided in Supplementary Data Sheets S1–S3.

##### Cilium assembly model

**FIGURE 5:**
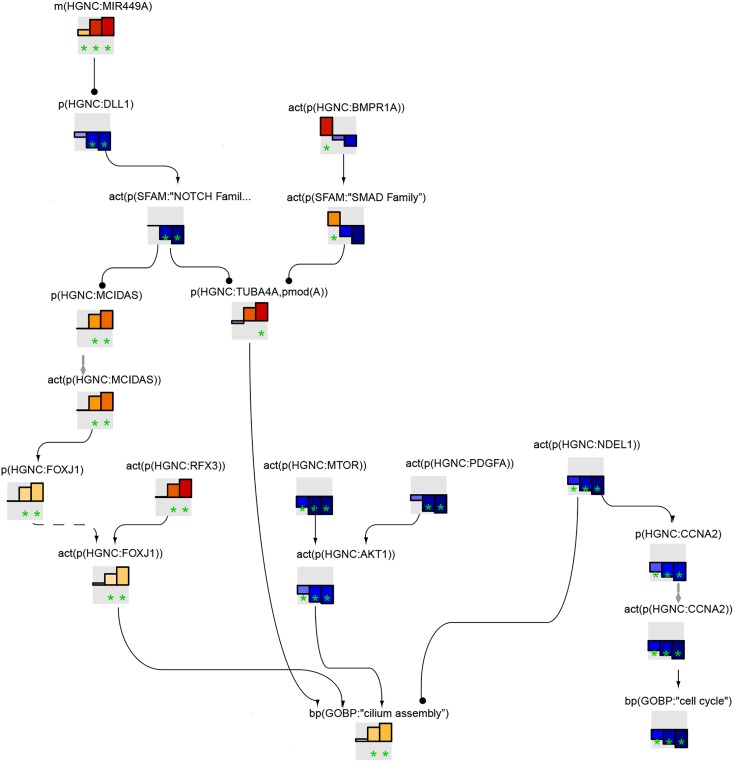
Cilium assembly subnetwork based on leading nodes. The graphical representation shows an example of connected leading nodes common for early (first lane), intermediate (second lane), and late (third lane) time points of human airway epithelial cell mucociliary differentiation. The top 10 leading nodes were prioritized and connected with other leading nodes from the analysis. The backbone NPA values with directionalities of inferred regulation are shown as bar graphs for each node. Orange/red bars indicate inferred upregulation and blue bars indicate inferred downregulation. The green asterisk indicates that the node is a leading node. The vocabulary for the BEL is provided in http://www.openbel.org/.

Figure 5 shows the leading node analysis of the cilium assembly network model scored with transcriptomic data from early, intermediate, and late time points of human airway epithelial cell mucociliary differentiation. At the early time point, bone morphogenic protein (BMP) signaling was inferred to be upregulated. The mechanistic target of rapamycin (mTOR), platelet-derived growth factor A (PDGFA), and protein kinase B (AKT) signaling were inferred to be downregulated, in contrast with the inferred upregulation of cilium assembly. At the same time, NudE neurodevelopment protein 1 like 1 (NDEL1) was inferred to be downregulated, resulting in downregulation of cyclin A2 (CCNA2) and cell cycle arrest. At the intermediate and late time points of mucociliary differentiation, BMP signaling was no longer inferred to be upregulated. Instead, DLL1/NOTCH1 signaling was inferred to be downregulated, resulting in an increase in MCIDAS and FOXJ1, the master transcription factors required for the formation of motile cilia. RFX3, also known to induce FOXJ1, was inferred to be upregulated at the intermediate and late time points. PDGFA, mTOR, AKT, and NDEL1/CCNA2 continued to be downregulated in the leading node analysis.

The leading node analysis of the pyocyanin treatment data scored on the cilium assembly network model indicated the upregulation of PDGFA and downregulation of BMP signaling (Supplementary Data Sheet S1).

##### Ciliary beating model

**FIGURE 6:**
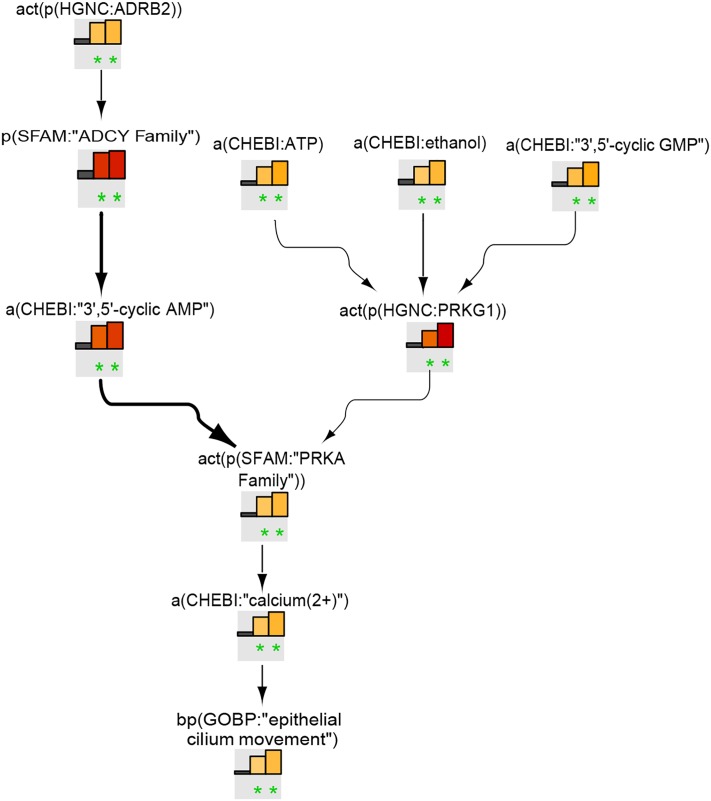
Ciliary beating subnetwork based on leading nodes. The graphical representation shows an example of connected leading nodes common for early (first lane), intermediate (second lane), and late (third lane) time points of human airway epithelial cell mucociliary differentiation. The top 10 leading nodes were prioritized and connected with other leadin g nodes from the analysis. The backbone NPA values with directionalities of inferred regulation are shown as bar graphs for each node. Orange/red bars indicate inferred upregulation and blue bars indicate inferred downregulation. Bold edges indicate “direct regulation.” The green asterisk indicates that the node is a leading node. The vocabulary for the BEL is provided in http://www.openbel.org/.

Figure 6 shows the leading node analysis of the ciliary beating network model scored with transcriptomic data from early, intermediate, and late time points of human airway epithelial cell mucociliary differentiation. The β2-adrenergic receptor/ADCY signaling pathway, leading to an increase in cAMP levels and subsequent Ca^2+^ increase via the activation of the PRKA family, was inferred to be upregulated. The analysis also inferred the activation of cGMP-dependent protein kinase 1 (PRKG1). In addition, the leading node analysis of the pyocyanin treatment data scored on the ciliary beating network model indicated the upregulation of ADCY and calcium signaling (Supplementary Data Sheet S2).

##### Goblet cell hyperplasia/metaplasia model

**FIGURE 7:**
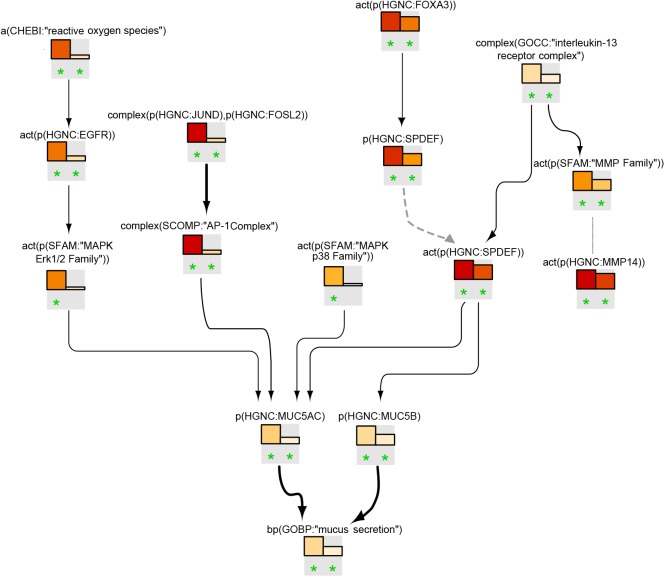
Goblet cell hyperplasia/metaplasia subnetwork based on leading nodes. The graphical representation shows an example of connected leading nodes for pyocyanin treatment (first lane) and IL13 treatment (second lane) compared with experimental controls. The top 10 leading nodes were prioritized and connected with other leading nodes from the analysis. Orange/red bars indicate inferred upregulation. Bold edges indicate “direct regulation.” The dotted edge denotes a member of a protein family. The green asterisk indicates that the node is a leading node in a given contrast. The vocabulary for the BEL is provided in http://www.openbel.org/.

Figure 7 shows the leading-node analysis of the goblet cell hyperplasia/metaplasia model with the pyocyanin and IL13 datasets. The levels of reactive oxygen species (ROS) were inferred to increase with subsequent activation of EGFR and ERK1/2, followed by an inferred increase in mucin production. Three other branches of the network that were highlighted and led to increases in mucins included the AP-1, FOXA3/SPDEF, and IL13/SPDEF pathways. The inferred activation of the IL13 receptor complex mirrored the activation of the matrix metalloproteinase family. The inferred activation of the p38 MAPK family upstream of MUC5AC was unique to the pyocyanin dataset. While the NPA analysis showed a significant network perturbation in response to human airway epithelial cell mucociliary differentiation, a closer examination of the leading nodes clearly indicated inferred downregulation of ROS and the EGFR and MAPK ERK1/2 pathways (Supplementary Data Sheet S3).

## Discussion

MCC is an important defense mechanism that protects the respiratory tract, and thus the body, from infections and airborne pollutants. In this article, we presented a suite of CBN models that describe relevant molecular processes related to MCC. Derived from original articles, the BEL-scripted scientific statements were assembled into three separate network models that capture molecular processes involved in cilium assembly, ciliary beating, and goblet cell hyperplasia/metaplasia accurately. The key factors involved in these processes are part of the backbone that interconnects various entities in the network models. As an example, the cilium assembly hub integrates the diversity of the cascades that are determined by the variety of cilia types, each requiring precise regulation (for review, see Choksi et al., 2014).

As part of network model validation, we conducted network scoring with gene expression data from experiments that were expected to trigger perturbation of the MCC models. The scoring also allowed us to look farther from the static network view into the key factors that impact the network and assess the behavior (activation or inhibition) of molecular entities in the model backbone based on differential gene expression in the selected datasets.

As expected, the biology in the redox-active pyocyanin treatment experiment was best reflected in the goblet cell hyperplasia/metaplasia model, with EGFR and downstream MAPK ERK1/2 factors predicted to be activated, leading to mucin production. This was in line with other experimental observations of increased numbers of goblet cells and increased mucin production in response to pyocyanin treatment (Rada et al., 2011).

Impact on the cilium-focused network models could be explained by the cell redox state and ROS levels affecting multiple cellular signaling pathways, some of which overlap with cilium biology. As an example, the activity of the nitric oxide synthase (NOS) family as well as the nitric oxide (NO) chemical node were inferred to be upregulated by pyocyanin treatment in the ciliary beating network model (Supplementary Data Sheet S2). NO is a redox molecule that regulates tissue oxidative balance through direct and indirect mechanisms of action and can lead to an increase in ciliary beat frequency through the activation of NOS family.

Network scoring with the airway epithelial cell differentiation dataset clearly showed time-dependent activation of pathways leading to cilium assembly and ciliary beating. At the early stage, BMP signaling was inferred to be upregulated, indicating the lack of cilium assembly, while at later time points, BMP released the inhibitory effect on cilium assembly, with the MCIDAS/FOXJ1/RFX3 pathway inferred to be activated to promote cilium assembly. Network scoring, however, indicated that mTOR and AKT were downregulated in the dataset, contradictory to the causal connection from mTOR and AKT to cilium assembly that was inferred to be upregulated. These relationships were derived from articles describing primary cilium assembly and may not appropriately reflect the biology in the respiratory tract, where the operating process is the motile cilia assembly program that culminates in the FOXJ1/RFX3 module (Wang et al., 2015; Suizu et al., 2016).

Downregulation of CCNA2 and cell cycle arrest in the cilium assembly network model could indicate a slowing down in cell proliferation to enforce cell differentiation. This was in accordance with the inferred inhibition of the mTOR/AKT pathway in the cilium assembly model. The results were further enforced by the scoring of the goblet cell network. The inferred reduction in EGFR signaling could indicate loss of proliferative potential in cultures differentiating to pseudostratified epithelium. This result also suggests that the network model discriminates between a physiological (i.e., differentiation) and pathological (i.e., COPD-related) increase in the number of goblet cells in airways. Finally, the cilia beating model appropriately captured the activation of cAMP/PRKA and cGMP/PRKG signaling that elevates cellular Ca^2+^ levels, leading to increases in cilia beating.

Scoring the three network models with the datasets from IL13-treated lung cells highlighted the specificities of the different networks: IL13-induced airway mucus production affected several hubs in the hyperplasia/metaplasia model, notably the SPDEF transcription factor, while impacts on the cilium assembly and ciliary beating models did not reach statistical significance.

In conclusion, the representation of cilium assembly, ciliary beating, and airway remodeling processes through CBN models is a potential powerful tool for systems medicine (Talikka et al., 2017). MCC networks can be used as a substrate for scoring high-throughput data for mechanistic understanding of the differences between diseased and healthy tissue. The MCC network model suite presented here, along with gene expression data from well-controlled clinical studies, could be used in individuals with MCC disorders for subject classification, identification of mode of action of novel drug candidates, or prediction of treatment outcome. Ultimately, the MCC network model suite provides perspectives for tailored drug therapy and precision medicine.

## Author Contributions

MT, JH, and MP conceived and designed the experiments. HY, SV, and MT performed the study. HY and MT wrote the manuscript with the support of KL. FM, AS, and SG analyzed the data. All authors made critical revision and approved the final version of the manuscript.

## Conflict of Interest Statement

All authors are employees of Philip Morris International.

## Footnotes

1 http://openbel.org/

2 http://www.cytoscape.org/

## Supplementary Material

DATA SHEET S1 Leading node analysis for cilium assembly network model scored with transcriptomic data from pyocyanin treatment, IL-13 treatment, and mucociliary differentiation datasets. For each node, an ^∗^ indicates it is a leading node and the number denotes its rank among the leading nodes. + or - in parenthesis indicates inferred up- or down-regulation, respectively. Value in % is the contribution of the node to the overall NPA.

DATA SHEET S2 Leading node analysis for ciliary beating network model scored with transcriptomic data from pyocyanin treatment, IL-13 treatment, and mucociliary differentiation datasets. For each node, an ^∗^ indicates it is a leading node and the number denotes its rank among the leading nodes. + or - in parenthesis indicates inferred up- or down-regulation, respectively. Value in % is the contribution of the node to the overall NPA.

DATA SHEET S3 Leading node analysis for goblet cell hyperplasia/metaplasia network model scored with transcriptomic data from pyocyanin treatment, IL-13 treatment, and mucociliary differentiation datasets. For each node, an ^∗^ indicates it is a leading node and the number denotes its rank among the leading nodes. + or - in parenthesis indicates inferred up- or down-regulation, respectively. Value in % is the contribution of the node to the overall NPA.

## References

[B1] Alevy Y. G. Patel A. C. Romero A. G. Patel D. A. Tucker J. Roswit W. T. (2012). IL-13–induced airway mucus production is attenuated by MAPK13 inhibition. *J. Clin. Invest.* 122 4555–4568. 10.1172/JCI64896 23187130 PMC3533556

[B2] Anderson D. Macnee W. (2009). Targeted treatment in COPD: a multi-system approach for a multi-system disease. *Int. J. Chron. Obstruct. Pulmon. Dis.* 4 321–335. 10.2147/COPD.S2999 19750192 PMC2740954

[B3] Arbi M. Pefani D. E. Kyrousi C. Lalioti M. E. Kalogeropoulou A. Papanastasiou A. D. (2016). GemC1 controls multiciliogenesis in the airway epithelium. *EMBO Rep.* 17 400–413. 10.15252/embr.201540882 26882546 PMC4772991

[B4] Blyth D. I. Pedrick M. S. Savage T. J. Bright H. Beesley J. E. Sanjar S. (1998). Induction, duration, and resolution of airway goblet cell hyperplasia in a murine model of atopic asthma: effect of concurrent infection with respiratory syncytial virus and response to dexamethasone. *Am. J. Respir. Cell Mol. Biol.* 19 38–54. 10.1165/ajrcmb.19.1.2930 9651179

[B5] Boon M. Wallmeier J. Ma L. Loges N. T. Jaspers M. Olbrich H. (2014). MCIDAS mutations result in a mucociliary clearance disorder with reduced generation of multiple motile cilia. *Nat. Commun.* 5:4418. 10.1038/ncomms5418 25048963

[B6] Boucherat O. Boczkowski J. Jeannotte L. Delacourt C. (2013). Cellular and molecular mechanisms of goblet cell metaplasia in the respiratory airways. *Exp. Lung Res.* 39 207–216. 10.3109/01902148.2013.791733 23638644

[B7] Boue S. Talikka M. Westra J. W. Hayes W. Di Fabio A. Park J. (2015). Causal biological network database: a comprehensive platform of causal biological network models focused on the pulmonary and vascular systems. *Database* 2015:bav030. 10.1093/database/bav030 25887162 PMC4401337

[B8] Brody S. L. Yan X. H. Wuerffel M. K. Song S. K. Shapiro S. D. (2000). Ciliogenesis and left-right axis defects in forkhead factor HFH-4-null mice. *Am. J. Respir. Cell Mol. Biol.* 23 45–51. 10.1165/ajrcmb.23.1.4070 10873152

[B9] Casalino-Matsuda S. M. Monzon M. E. Forteza R. M. (2006). Epidermal growth factor receptor activation by epidermal growth factor mediates oxidant-induced goblet cell metaplasia in human airway epithelium. *Am. J. Respir. Cell Mol. Biol.* 34 581–591. 10.1165/rcmb.2005-0386OC 16424381 PMC2644222

[B10] Catlett N. L. Bargnesi A. J. Ungerer S. Seagaran T. Ladd W. Elliston K. O. (2013). Reverse causal reasoning: applying qualitative causal knowledge to the interpretation of high-throughput data. *BMC Bioinformatics* 14:340. 10.1186/1471-2105-14-340 24266983 PMC4222496

[B11] Chen G. Korfhagen T. R. Xu Y. Kitzmiller J. Wert S. E. Maeda Y. (2009). SPDEF is required for mouse pulmonary goblet cell differentiation and regulates a network of genes associated with mucus production. *J. Clin. Invest.* 119 2914–2924. 10.1172/JCI39731 19759516 PMC2752084

[B12] Chen J. Knowles H. J. Hebert J. L. Hackett B. P. (1998). Mutation of the mouse hepatocyte nuclear factor/forkhead homologue 4 gene results in an absence of cilia and random left-right asymmetry. *J. Clin. Invest.* 102 1077–1082. 10.1172/JCI4786 9739041 PMC509090

[B13] Cho D. Y. Kim Y. A. Przytycka T. M. (2012). Chapter 5: network biology approach to complex diseases. *PLoS Comput. Biol.* 8:e1002820. 10.1371/journal.pcbi.1002820 23300411 PMC3531284

[B14] Choksi S. P. Lauter G. Swoboda P. Roy S. (2014). Switching on cilia: transcriptional networks regulating ciliogenesis. *Development* 141 1427–1441. 10.1242/dev.074666 24644260

[B15] De Leon H. Boue S. Schlage W. K. Boukharov N. Westra J. W. Gebel S. (2014). A vascular biology network model focused on inflammatory processes to investigate atherogenesis and plaque instability. *J. Transl. Med.* 12:185. 10.1186/1479-5876-12-185 24965703 PMC4227037

[B16] Fernandes J. Lorenzo I. M. Andrade Y. N. Garcia-Elias A. Serra S. A. Fernandez-Fernandez J. M. (2008). IP3 sensitizes TRPV4 channel to the mechano- and osmotransducing messenger 5′-6′-epoxyeicosatrienoic acid. *J. Cell Biol.* 181 143–155. 10.1083/jcb.200712058 18378772 PMC2287294

[B17] Fliegauf M. Benzing T. Omran H. (2007). When cilia go bad: cilia defects and ciliopathies. *Nat. Rev. Mol. Cell Biol.* 8 880–893. 10.1038/nrm2278 17955020

[B18] Gebel S. Lichtner R. B. Frushour B. Schlage W. K. Hoang V. Talikka M. (2013). Construction of a computable network model for DNA damage, autophagy, cell death, and senescence. *Bioinform. Biol. Insights* 7 97–117. 10.4137/BBI.S11154 23515068 PMC3596057

[B19] Gerdes J. M. Davis E. E. Katsanis N. (2009). The vertebrate primary cilium in development, homeostasis, and disease. *Cell* 137 32–45. 10.1016/j.cell.2009.03.023 19345185 PMC3016012

[B20] Hao Y. Kuang Z. Jing J. Miao J. Mei L. Y. Lee R. J. (2014). Mycoplasma pneumoniae modulates STAT3-STAT6/EGFR-FOXA2 signaling to induce overexpression of airway mucins. *Infect. Immun.* 82 5246–5255. 10.1128/IAI.01989-14 25287927 PMC4249270

[B21] Hewson C. A. Edbrooke M. R. Johnston S. L. (2004). PMA induces the MUC5AC respiratory mucin in human bronchial epithelial cells, via PKC, EGF/TGF-alpha, Ras/Raf, MEK, ERK and Sp1-dependent mechanisms. *J. Mol. Biol.* 344 683–695. 10.1016/j.jmb.2004.09.059 15533438

[B22] Hoeng J. Deehan R. Pratt D. Martin F. Sewer A. Thomson T. M. (2012). A network-based approach to quantifying the impact of biologically active substances. *Drug Discov. Today* 17 413–418. 10.1016/j.drudis.2011.11.008 22155224

[B23] Hubbert C. Guardiola A. Shao R. Kawaguchi Y. Ito A. Nixon A. (2002). HDAC6 is a microtubule-associated deacetylase. *Nature* 417 455–458. 10.1038/417455a 12024216

[B24] Jain B. Rubinstein I. Robbins R. A. Leise K. L. Sisson J. H. (1993). Modulation of airway epithelial cell ciliary beat frequency by nitric oxide. *Biochem. Biophys. Res. Commun.* 191 83–88. 10.1006/bbrc.1993.1187 7680560

[B25] Kempeneers C. Chilvers M. A. (2018). To beat, or not to beat, that is question! The spectrum of ciliopathies. *Pediatr. Pulmonol.* 53 1122–1129. 10.1002/ppul.24078 29938933

[B26] Kim S. Tsiokas L. (2011). Cilia and cell cycle re-entry: more than a coincidence. *Cell Cycle* 10 2683–2690. 10.4161/cc.10.16.17009 21814045 PMC3219538

[B27] Kim V. Criner G. J. (2015). The chronic bronchitis phenotype in chronic obstructive pulmonary disease: features and implications. *Curr. Opin. Pulm. Med.* 21 133–141. 10.1097/MCP.0000000000000145 25575367 PMC4373868

[B28] Korngreen A. Priel Z. (1996). Purinergic stimulation of rabbit ciliated airway epithelia: control by multiple calcium sources. *J. Physiol.* 497(Pt 1), 53–66. 10.1113/jphysiol.1996.sp021749 8951711 PMC1160912

[B29] Luettich K. Talikka M. Lowe F. J. Haswell L. E. Park J. Gaca M. D. (2017). The adverse outcome pathway for oxidative stress-mediated EGFR activation leading to decreased lung function. *Appl. Vitro Toxicol.* 3 99–109. 10.1089/aivt.2016.0032

[B30] Martin F. Sewer A. Talikka M. Xiang Y. Hoeng J. Peitsch M. C. (2014). Quantification of biological network perturbations for mechanistic insight and diagnostics using two-layer causal models. *BMC Bioinformatics* 15:238. 10.1186/1471-2105-15-238 25015298 PMC4227138

[B31] Matera M. G. Calzetta L. Cazzola M. (2016). Oxidation pathway and exacerbations in COPD: the role of NAC. *Expert Rev. Respir. Med.* 10 89–97. 10.1586/17476348.2016.1121105 26567752

[B32] Murphy D. B. Seemann S. Wiese S. Kirschner R. Grzeschik K. H. Thies U. (1997). The human hepatocyte nuclear factor 3/fork head gene FKHL13: genomic structure and pattern of expression. *Genomics* 40 462–469. 10.1006/geno.1996.4587 9073514

[B33] Nini G. Raica M. Neamtiu V. Onel M. (2012). Morphological study of bronchial mucosa in the chronic obstructive pulmonary disease under the influence of therapeutic algorithm. *Rom. J. Morphol. Embryol.* 53 121–134. 22395511

[B34] Nozawa Y. I. Lin C. Chuang P. T. (2013). Hedgehog signaling from the primary cilium to the nucleus: an emerging picture of ciliary localization, trafficking and transduction. *Curr. Opin. Genet Dev.* 23 429–437. 10.1016/j.gde.2013.04.008 23725801 PMC3913210

[B35] Pan J. Wang Q. Snell W. J. (2004). An aurora kinase is essential for flagellar disassembly in Chlamydomonas. *Dev. Cell* 6 445–451. 10.1016/S1534-5807(04)00064-4 15030766

[B36] Park K. S. Korfhagen T. R. Bruno M. D. Kitzmiller J. A. Wan H. Wert S. E. (2007). SPDEF regulates goblet cell hyperplasia in the airway epithelium. *J. Clin. Invest.* 117 978–988. 10.1172/JCI29176 17347682 PMC1810569

[B37] Perrais M. Pigny P. Copin M. C. Aubert J. P. Van Seuningen I. (2002). Induction of MUC2 and MUC5AC mucins by factors of the epidermal growth factor (EGF) family is mediated by EGF receptor/Ras/Raf/extracellular signal-regulated kinase cascade and Sp1. *J. Biol. Chem.* 277 32258–32267. 10.1074/jbc.M204862200 12077147

[B38] Pugacheva E. N. Jablonski S. A. Hartman T. R. Henske E. P. Golemis E. A. (2007). HEF1-dependent Aurora A activation induces disassembly of the primary cilium. *Cell* 129 1351–1363. 10.1016/j.cell.2007.04.035 17604723 PMC2504417

[B39] Rada B. Gardina P. Myers T. G. Leto T. L. (2011). Reactive oxygen species mediate inflammatory cytokine release and EGFR-dependent mucin secretion in airway epithelial cells exposed to *Pseudomonas* pyocyanin. *Mucosal Immunol.* 4 158–171. 10.1038/mi.2010.62 20962773 PMC3026888

[B40] Rahman I. Adcock I. M. (2006). Oxidative stress and redox regulation of lung inflammation in COPD. *Eur. Respir. J.* 28 219–242. 10.1183/09031936.06.00053805 16816350

[B41] Rahman I. MacNee W. (1999). Lung glutathione and oxidative stress: implications in cigarette smoke-induced airway disease. *Am. J. Physiol.* 277 L1067–L1088. 10.1152/ajplung.1999.277.6.L1067 10600876

[B42] Ramos F. L. Krahnke J. S. Kim V. (2014). Clinical issues of mucus accumulation in COPD. *Int. J. Chron. Obstruct. Pulmon. Dis.* 9 139–150. 10.2147/COPD.S38938 24493923 PMC3908831

[B43] Rogers D. F. (2007). Physiology of airway mucus secretion and pathophysiology of hypersecretion. *Respir. Care* 52 1134–1146;discussion 1146–1139. 17716382

[B44] Ross A. J. Dailey L. A. Brighton L. E. Devlin R. B. (2007). Transcriptional profiling of mucociliary differentiation in human airway epithelial cells. *Am. J. Respir. Cell Mol. Biol.* 37 169–185. 10.1165/rcmb.2006-0466OC 17413031

[B45] Satir P. Christensen S. T. (2007). Overview of structure and function of mammalian cilia. *Annu. Rev. Physiol.* 69 377–400. 10.1146/annurev.physiol.69.040705.141236 17009929

[B46] Schlage W. K. Westra J. W. Gebel S. Catlett N. L. Mathis C. Frushour B. P. (2011). A computable cellular stress network model for non-diseased pulmonary and cardiovascular tissue. *BMC Syst. Biol.* 5:168. 10.1186/1752-0509-5-168 22011616 PMC3224482

[B47] Sewer A. Martin F. Schlage W. K. Hoeng J. Peitsch M. C. (2015). “Quantifying the Biological Impact of Active Substances Using Causal Network Models,” in *Computational Systems Toxicology*, eds Hoeng J. Peitsch M. (New York, NY: Humana Press), 223–256.

[B48] Shannon P. Markiel A. Ozier O. Baliga N. S. Wang J. T. Ramage D. (2003). Cytoscape: a software environment for integrated models of biomolecular interaction networks. *Genome Res.* 13 2498–2504. 10.1101/gr.1239303 14597658 PMC403769

[B49] Smyth G. K. (2004). Linear models and empirical bayes methods for assessing differential expression in microarray experiments. *Stat. Appl. Genet Mol. Biol.* 3:Article3. 10.2202/1544-6115.1027 16646809

[B50] Stubbs J. L. Vladar E. K. Axelrod J. D. Kintner C. (2012). Multicilin promotes centriole assembly and ciliogenesis during multiciliate cell differentiation. *Nat. Cell Biol.* 14 140–147. 10.1038/ncb2406 22231168 PMC3329891

[B51] Suizu F. Hirata N. Kimura K. Edamura T. Tanaka T. Ishigaki S. (2016). Phosphorylation-dependent Akt-Inversin interaction at the basal body of primary cilia. *EMBO J.* 35 1346–1363. 10.15252/embj.201593003 27220846 PMC4883026

[B52] Sutto Z. Conner G. E. Salathe M. (2004). Regulation of human airway ciliary beat frequency by intracellular pH. *J. Physiol.* 560 519–532. 10.1113/jphysiol.2004.068171 15308676 PMC1665258

[B53] Szostak J. Ansari S. Madan S. Fluck J. Talikka M. Iskandar A. (2015). Construction of biological networks from unstructured information based on a semi-automated curation workflow. *Database* 2015:bav057. 10.1093/database/bav057 26200752 PMC5630939

[B54] Szostak J. Martin F. Talikka M. Peitsch M. C. Hoeng J. (2016). Semi-automated curation allows causal network model building for the quantification of age-dependent plaque progression in ApoE-/- mouse. *Gene Regul. Syst. Bio.* 10 95–103. 10.4137/GRSB.S40031 27840576 PMC5100841

[B55] Takeyama K. Dabbagh K. Lee H. M. Agusti C. Lausier J. A. Ueki I. F. (1999). Epidermal growth factor system regulates mucin production in airways. *Proc. Natl. Acad. Sci. U.S.A.* 96 3081–3086. 10.1073/pnas.96.6.3081 10077640 PMC15898

[B56] Takeyama K. Jung B. Shim J. J. Burgel P. R. Dao-Pick T. Ueki I. F. (2001). Activation of epidermal growth factor receptors is responsible for mucin synthesis induced by cigarette smoke. *Am. J. Physiol. Lung Cell Mol. Physiol.* 280 L165–L172. 10.1152/ajplung.2001.280.1.L165 11133506

[B57] Talikka M. Bukharov N. Hayes W. S. Hofmann-Apitius M. Alexopoulos L. Peitsch M. C. (2017). Novel approaches to develop community-built biological network models for potential drug discovery. *Expert Opin. Drug Discov.* 12 849–857. 10.1080/17460441.2017.1335302 28585481

[B58] Thomas J. Morle L. Soulavie F. Laurencon A. Sagnol S. Durand B. (2010). Transcriptional control of genes involved in ciliogenesis: a first step in making cilia. *Biol. Cell* 102 499–513. 10.1042/BC20100035 20690903

[B59] Turner J. Jones C. E. (2009). Regulation of mucin expression in respiratory diseases. *Biochem. Soc. Trans.* 37 877–881. 10.1042/BST0370877 19614611

[B60] Wang S. Livingston M. J. Su Y. Dong Z. (2015). Reciprocal regulation of cilia and autophagy via the MTOR and proteasome pathways. *Autophagy* 11 607–616. 10.1080/15548627.2015.1023983 25906314 PMC4502771

[B61] Wanner A. Salathe M. O’Riordan T. G. (1996). Mucociliary clearance in the airways. *Am. J. Respir. Crit. Care Med.* 154 1868–1902. 10.1164/ajrccm.154.6.8970383 8970383

[B62] Westra J. W. Schlage W. K. Frushour B. P. Gebel S. Catlett N. L. Han W. (2011). Construction of a computable cell proliferation network focused on non-diseased lung cells. *BMC Syst. Biol.* 5:105. 10.1186/1752-0509-5-105 21722388 PMC3160372

[B63] Westra J. W. Schlage W. K. Hengstermann A. Gebel S. Mathis C. Thomson T. (2013). A modular cell-type focused inflammatory process network model for non-diseased pulmonary tissue. *Bioinform. Biol. Insights* 7 167–192. 10.4137/BBI.S11509 23843693 PMC3700945

[B64] Wheatley D. N. (1995). Primary cilia in normal and pathological tissues. *Pathobiology* 63 222–238. 10.1159/000163955 8866794

[B65] Wills-Karp M. (2004). Interleukin-13 in asthma pathogenesis. *Immunol. Rev.* 202 175–190. 10.1111/j.0105-2896.2004.00215.x 15546393

[B66] Workman A. D. Cohen N. A. (2014). The effect of drugs and other compounds on the ciliary beat frequency of human respiratory epithelium. *Am. J. Rhinol. Allergy* 28 454–464. 10.2500/ajra.2014.28.4092 25514481

[B67] Wyatt T. A. Spurzem J. R. May K. Sisson J. H. (1998). Regulation of ciliary beat frequency by both PKA and PKG in bovine airway epithelial cells. *Am. J. Physiol.* 275 L827–L835. 10.1152/ajplung.1998.275.4.L827 9755116

[B68] Yaghi A. Dolovich M. B. (2016). Airway Epithelial Cell Cilia and Obstructive Lung Disease. *Cells* 5:40. 10.3390/cells5040040 27845721 PMC5187524

[B69] Yaghi A. Zaman A. Cox G. Dolovich M. B. (2012). Ciliary beating is depressed in nasal cilia from chronic obstructive pulmonary disease subjects. *Respir. Med.* 106 1139–1147. 10.1016/j.rmed.2012.04.001 22608352

[B70] Yang B. Schlosser R. J. McCaffrey T. V. (1996). Dual signal transduction mechanisms modulate ciliary beat frequency in upper airway epithelium. *Am. J. Physiol.* 270 L745–L751. 10.1152/ajplung.1996.270.5.L745 8967508

[B71] Zagoory O. Braiman A. Gheber L. Priel Z. (2001). Role of calcium and calmodulin in ciliary stimulation induced by acetylcholine. *Am. J. Physiol. Cell Physiol.* 280 C100–C109. 10.1152/ajpcell.2001.280.1.C100 11121381

[B72] Zagoory O. Braiman A. Priel Z. (2002). The mechanism of ciliary stimulation by acetylcholine: roles of calcium, PKA, and PKG. *J. Gen. Physiol.* 119 329–339. 10.1085/jgp.20028519 11929884 PMC2311390

