# Erratum: Construction of a Suite of Computable Biological Network Models Focused on Mucociliary Clearance in the Respiratory Tract

**DOI:** 10.3389/fgene.2020.00100

**Published:** 2020-02-07

**Authors:** Frontiers Production Office

**Affiliations:** Frontiers Media SA, Lausanne, Switzerland

**Keywords:** mucociliary clearance, network models, biological expression language, respiratory tract, network perturbation amplitude

Due to a typesetting error, **Figure 2** was duplicated, and the correct **Figure 1** was not included in the original article. The correct **Figure 1** and caption are included in this article.

**Figure 1:**
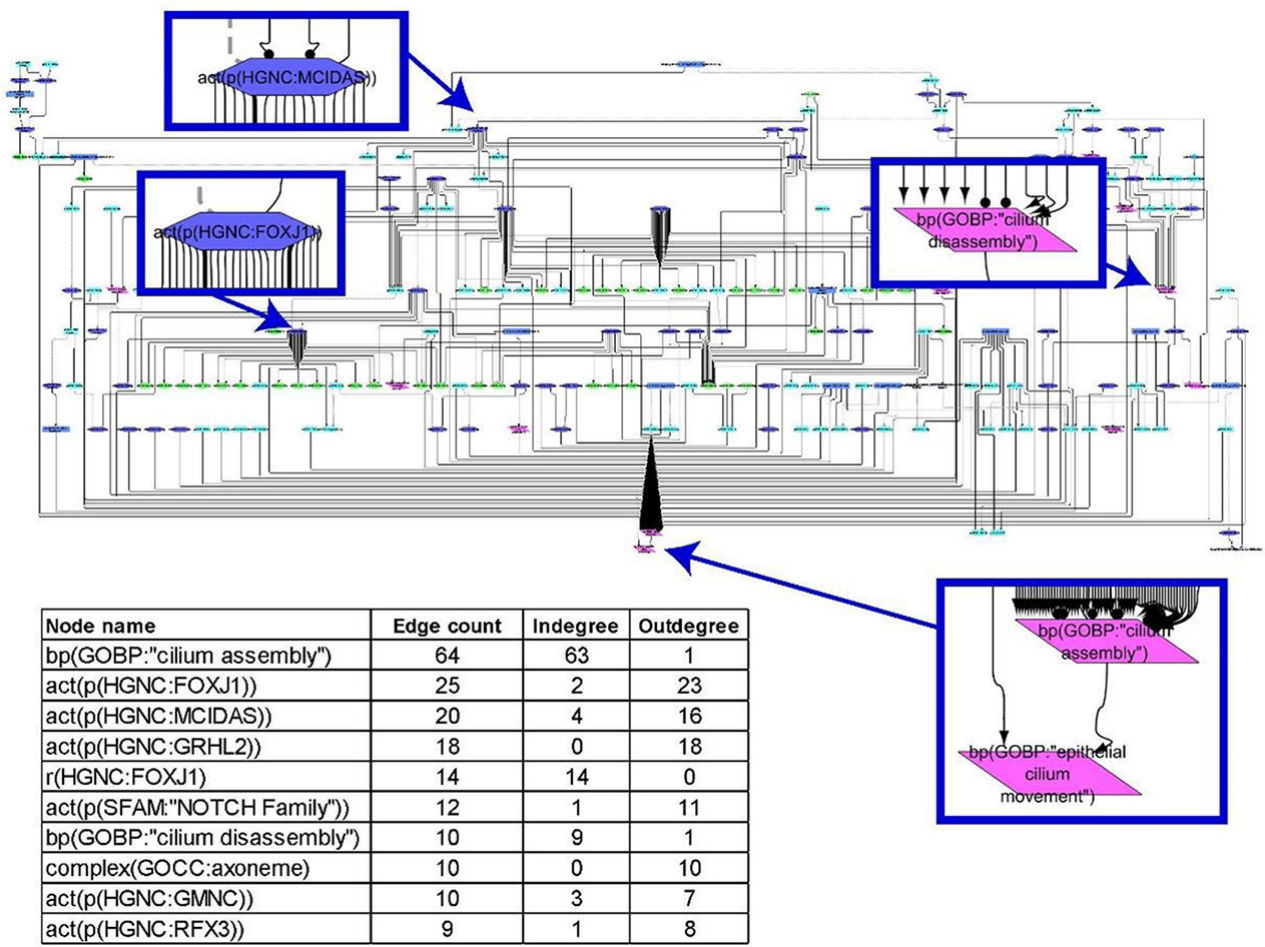
Causal biological network model for cilium assembly. The table shows the top 10 highly connected nodes and their degrees of distribution. The vocabulary for the BEL is provided in http://www.openbel.org/. The Cytoscape layout is the Yfiles hierarchical layout. The network model can be downloaded from causalbionet.com.

The original version of this article has been updated.

